# Non-linear Relationship of Maternal Age With Risk of Spontaneous Abortion: A Case-Control Study in the China Birth Cohort

**DOI:** 10.3389/fpubh.2022.933654

**Published:** 2022-07-14

**Authors:** Man Zhang, Bo-Yi Yang, Yongqing Sun, Zhengmin Qian, Pamela K. Xaverius, Hannah E. Aaron, Xiaoting Zhao, Zheng Zhang, Ruixia Liu, Guang-Hui Dong, Chenghong Yin, Wentao Yue

**Affiliations:** ^1^Central Laboratory, Beijing Obstetrics and Gynecology Hospital, Capital Medical University, Beijing, China; ^2^Beijing Maternal and Child Health Care Hospital, Beijing, China; ^3^Guangzhou Key Laboratory of Environmental Pollution and Health Risk Assessment, Guangdong Provincial Engineering Technology Research Center of Environmental and Health Risk Assessment, Department of Preventive Medicine, School of Public Health, Sun Yat-sen University, Guangzhou, China; ^4^Prenatal Diagnosis Center, Beijing Obstetrics and Gynecology Hospital, Capital Medical University, Beijing, China; ^5^Department of Epidemiology and Biostatistics, College for Public Health and Social Justice, Saint Louis University, Saint Louis, MO, United States

**Keywords:** non-linear relationship, maternal age, spontaneous abortion, case-control, China Birth Cohort

## Abstract

**Background:**

Spontaneous abortion is one of the prevalent adverse reproductive outcomes, which seriously threatens maternal health around the world.

**Objective:**

The current study is aimed to evaluate the association between maternal age and risk for spontaneous abortion among pregnant women in China.

**Methods:**

This was a case-control study based on the China Birth Cohort, we compared 338 cases ending in spontaneous abortion with 1,352 controls resulting in normal live births. The main exposure indicator and outcome indicator were maternal age and spontaneous abortion, respectively. We used both a generalized additive model and a two-piece-wise linear model to determine the association. We further performed stratified analyses to test the robustness of the association between maternal age and spontaneous abortion in different subgroups.

**Results:**

We observed a J-shaped relationship between maternal age and spontaneous abortion risk, after adjusting for multiple covariates. Further, we found that the optimal threshold age was 29.68 years old. The adjusted odds ratio (95% confidence interval) of spontaneous abortion per 1 year increase in maternal age were 0.97 (0.90–1.06) on the left side of the turning point and 1.25 (1.28–1.31) on the right side. Additionally, none of the covariates studied modified the association between maternal age and spontaneous abortion (*P* > 0.05).

**Conclusions:**

Advanced maternal age (>30 years old) was significantly associated with increased prevalence of spontaneous abortion, supporting a J-shaped association between maternal age and spontaneous abortion.

## Introduction

Spontaneous abortion is defined as fetal loss before 28 gestational weeks (1). Among all pregnant women, the incidence of spontaneous abortion is rising and it is the most common complication in the first trimester (2–4). The risk of adverse maternal and infant outcomes increases due to advanced maternal age (5). Studies have found that advanced maternal age can increase the risk of maternal conditions such as gestational diabetes, gestational hypertension, and preeclampsia (6). Advanced maternal age was also associated with an increased risk of spontaneous abortion, ectopic pregnancy, perinatal mortality, preterm birth, low birth weight, and intrauterine growth restriction (7). So, an accurate assessment and reporting of the association between maternal age and risk for spontaneous abortion is vital to the research and surveillance of maternal and infant health (8, 9).

Studies on the relationship between maternal age and spontaneous abortion have yielded inconsistent results (10–12). Some studies observed that advanced maternal age was associated with an increased risk of spontaneous abortion (13, 14), while others have shown that low maternal age is a risk factor for spontaneous abortion (15, 16). Ben-David et al. (12) suggested that maternal age and spontaneous abortion may not have an obvious relationship. Magnus et al. (17) reported that there is a J-shaped relationship between maternal age and spontaneous abortion. The threshold maternal age for spontaneous abortion ranged from 20 to 45 years old in various studies (16–19). However, the majority of studies categorized participants as either above or below 30 or 35 years old, which limited the comparability between scientific and clinical guidance given by different studies. Most domestic studies concentrated on establishing a linear relationship between maternal age and risk of spontaneous abortion or recurrent miscarriage, and only two small-sample studies discussed a non-linear relationship between maternal age and risk of spontaneous abortion, finding that the risk is slightly elevated in the youngest mothers and then rose sharply in mothers 32 years and older (20, 21). The precise form of the non-linear relationship between maternal age and spontaneous abortion risk is unclear, nor is there any discussion of a threshold effect of that.

Nowadays, more and more women delay getting married and having babies for educational, social, and economic reasons, the situation could trigger a series of health problems including miscarriages, birth defects (22), and the offspring health (23). A safe maternal age for pregnant women is the main foundation of sustained human development and a top priority for the international community (24). Given that prior research produced conflicting results, we selected a case-control sample nested within a large Chinese birth cohort to assess the relationship between maternal age and spontaneous abortion. Furthermore, we investigated the shape of the exposure-response curve of the association.

## Methods

### Study Design and Participants

We conducted a matched case-control study that was nested within the China Birth Cohort Study (CBCS), which is a populated-based birth defects surveillance system in China between November 2017 and December 2021 (25). The CBCS was conducted in 38 research sites in 17 provinces, cities, autonomous regions and municipalities covering most areas of China. We obtained data access permissions for 4 hospitals from them. Pregnant women (*n* = 8,873) were initially recruited to this study during their first prenatal visit in the four hospitals from January 2021 to December 2021. Exclusion criteria included multiple pregnancies (*n* = 204), participants with missing data for birth outcome (*n* = 37), with missing data for maternal age (*n* = 17), with age not between 18 and 45 years (*n* = 9), with severe mental disorders (*n* = 55). Thus, 8,551 singleton pregnancies were remained (581 participants with abortion and 7,970 controls). We further excluded participants with unnatural abortions (*n* = 243) for the case group, and participants with adverse pregnancy outcomes (including small for gestational age, large for gestational age, macrosomia, cesarean delivery, gestational diabetes mellitus, preeclampsia, postpartum weight retention, and offspring obesity) (26).

**Figure 1:**
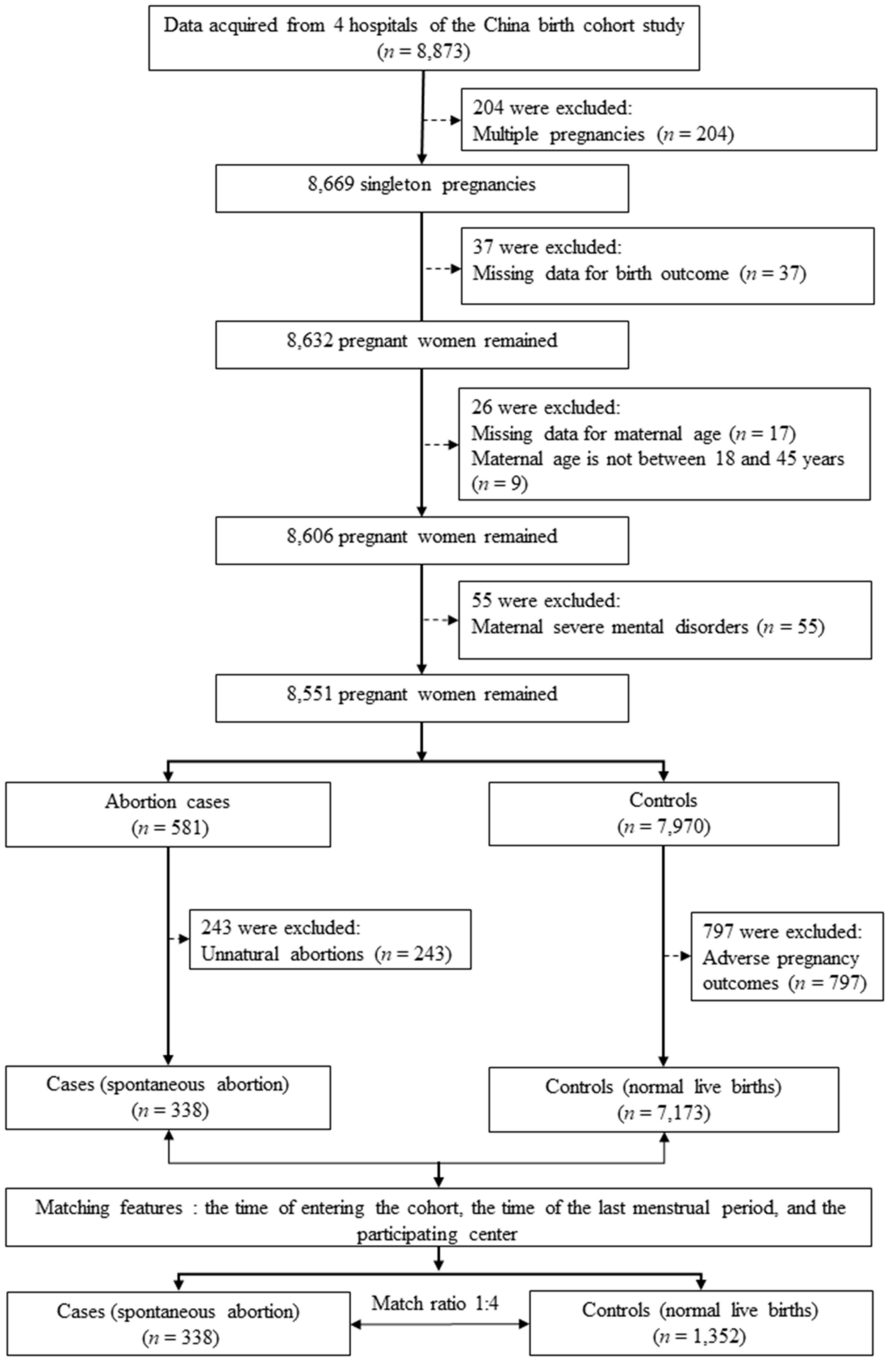
Flow chart of study participants selection.

We matched four controls for each case (27, 28). To retain cases as much as possible and avoid the reduced power caused by unnecessary matching variables (29), we matched controls based on: (a) the time of entering the cohort to reduce biases from environment factors (30); (b) the time of the last menstrual period, because the prevalence of spontaneous abortion is higher in the first trimester (31); and (c) the participating center, the difference in technical levels in different medical centers may affect the relationship between maternal age and risk of spontaneous abortion (27). The final study population included 338 cases (*i.e.*, all participants with spontaneous abortion during the study period) and 1,352 controls (*i.e.*, participants with normal live births) (Figure 1).

### Variables and Data Measurement

Our exposure of interest was maternal age, which was calculated as (date of last menstrual period minus maternal birthdate)/365.25 (32). Our outcome was spontaneous abortion which refers to the loss of a clinically recognized pregnancy before the 28th week estimated gestational age (33).

We identified potential covariates using a literature search. Most of the covariate data were obtained from standard and structured questionnaires: maternal ethnicity (Han/minority) (17), maternal education (below college/college/college graduate) (34), maternal occupation (manual/non-manual/unemployed) (35), maternal income (<50,000 yuan/50,000–100,000 yuan/>100,000 yuan) (34), parity (nullipara/multipara) (36), conception method (natural conception/assisted reproduction) (20), maternal secondhand smoking (no/yes) (37), maternal drinking (no/yes), folic acid supplementation (no/yes) (38), multivitamins supplementation (no/yes) (39), medication use (no/yes) (40). The height and weight of the pregnant women were obtained through accurate measurement. The value of weight was accurately measured using an electronic scale (BW-150; UWE, Beijing, China) with participants wearing light clothes, no shoes, and empty pockets (41). Standing height was measured to the nearest 0.1 cm using a stadiometer (42). Body mass index (BMI) was calculated by dividing body weight (kg) by square of height (m).

### Statistical Analysis

Data are given as mean ± standard deviations (SD), median (interquartile range), or frequency (%), as appropriate. We used χ^2^ test or Kruskal-Wallis test to compare case and control groups. To investigate the independent correlation between maternal age and spontaneous abortion, three multivariate logistic regression models either unadjusted, minimally adjusted or fully adjusted for confounding factors were constructed at the same time. To identify the non-linearity between maternal age and spontaneous abortion, generalized additive models (GAM) were used. If a non-linear correlation was detected, a 2-piecewise logistic regression model was performed to calculate the threshold effect of maternal age on risk of spontaneous abortion regarding the smoothing plot. When the ratio between maternal age and spontaneous abortion appears obvious on the smoothed curve, a recursive method automatically calculated the inflection point, using the maximum model likelihood (43). Finally, to test the robustness of the association between maternal age and spontaneous abortion, we further conducted stratified analyses based on subgroups defined by the main covariates known to affect spontaneous abortion risk, including maternal ethnicity, maternal income, maternal BMI, parity, folic acid supplementation, and multivitamins supplementation, except for the stratification factor itself. All statistical analyses were completed using R version 4.1.1 (http://www.R-project.org).

## Results

### Characteristics of Cases and Controls

Table 1 shows characteristics of the 338 cases and 1,352 controls. Compared with participants without spontaneous abortion, pregnant women resulting in spontaneous abortion were more likely to be older (32.12 vs. 29.28 years), nullipara (60.36 vs. 48.89%), to have higher BMI (22.89 vs. 21.29 kg/m^2^), to possess income higher than 100,000 yuan (38.17 vs. 26.78%), to take medication during pregnancy (45.56 vs. 38.54%), as well as who had not taken folic acid (9.47 vs. 6.14%).

**Table 1:** Study characteristics of cases and controls.

**Variables**	**Control (*n* = 1,352)**	**Case (*n* = 338)**	***P-*value**
Maternal age (mean ± SD), years	29.28 ± 3.79	32.12 ± 4.71	<0.001
Maternal BMI (mean ± SD), kg/m^2^	21.29 ± 3.11	22.89 ± 4.14	<0.001
**Maternal ethnicity**, ***n*** **(%)**			0.032
Han	1,315 (97.26%)	321 (94.97%)	
Minority	37 (2.74%)	17 (5.03%)	
**Maternal education**, ***n*** **(%)**			0.003
College graduate	88 (6.51%)	39 (11.54%)	
College	545 (40.31%)	143 (42.31%)	
Below college	719 (53.18%)	156 (46.15%)	
**Maternal occupation**, ***n*** **(%)**			0.022
Non-manual	362 (26.77%)	85 (25.15%)	
Manual	627 (46.38%)	183 (54.14%)	
Unemployed	363 (26.85%)	70 (20.71%)	
**Secondhand smoking**, ***n*** **(%)**			0.663
No	1,186 (87.72%)	293 (86.69%)	
Yes	162 (11.98%)	43 (12.72%)	
Missing	4 (0.30%)	2 (0.59%)	
**Maternal drinking**, ***n*** **(%)**			0.832
No	1,289 (95.34%)	321 (94.97%)	
Yes	62 (4.59%)	17 (5.03%)	
Missing	1 (0.07%)	0 (0.00%)	
**Maternal income, yuan**, ***n*** **(%)**			<0.001
>100,000	362 (26.78%)	129 (38.17%)	
50,000–100,000	593 (43.86%)	120 (35.50%)	
<50,000	397 (29.36%)	89 (26.33%)	
**Parity**, ***n*** **(%)**			<0.001
Nullipara	661 (48.89%)	204 (60.36%)	
Multipara	691 (51.11%)	134 (39.64%)	
**Conception method**, ***n*** **(%)**			0.462
Natural conception	1,289 (95.34%)	319 (94.38%)	
Assisted reproduction	63 (4.66%)	19 (5.62%)	
**Folic acid supplementation**, ***n*** **(%)**			0.030
No	83 (6.14%)	32 (9.47%)	
First trimester of pregnancy	810 (59.91%)	181 (53.55%)	
Before pregnancy	459 (33.95%)	125 (36.98%)	
**Multivitamin supplementation**, ***n*** **(%)**			<0.001
No	626 (46.30%)	110 (32.54%)	
First trimester of pregnancy	540 (39.94%)	140 (41.42%)	
Before pregnancy	186 (13.76%)	88 (26.04%)	
**Medication use**, ***n*** **(%)**			0.018
No	831 (61.46%)	184 (54.44%)	
Yes	521 (38.54%)	154 (45.56%)	

*SD, standard deviation; BMI, body mass index*.

### Association of Maternal Age With Spontaneous Abortion

Table 2 shows the independent role of maternal age on spontaneous abortion. In model 3 (*i.e.*, fully adjusted model) (covariates test in Supplementary Table S1) each 1-year increase in maternal age was associated with a 16% increased risk of spontaneous abortion, and results were statistically significant. For sensitivity analyses, we also handled maternal age as a categorical variable (through division into tertiles). Participants in the high age group (31.30–44.92 years) had a significantly increased risk of spontaneous abortion [adjusted odds ratio (aOR) = 2.90; 95% confidence interval (CI): 2.02–4.15] in comparison to participants in the low age group (18.73–28.01 years). Spontaneous abortion was not statistically significant in the middle age group (28.02–31.29 years) (aOR = 1.22; 95% CI: 0.84–1.77). These results suggest that the association between maternal age and spontaneous abortion is likely to be non-linear.

**Table 2:** Multivariate logistic regression analysis for the association between maternal age and risk of spontaneous abortion.

**Variables**	**Model 1 OR (95% CI)**	**Model 2 aOR (95% CI)**	**Model 3 aOR (95% CI)**
Maternal age (continuous), years	1.18 (1.15–1.22)	1.18 (1.14–1.22)	1.16 (1.12–1.20)
**Maternal age (tertiles), years**
Low (18.73–28.01)	Reference	Reference	Reference
Middle (28.02–31.29)	1.66 (1.17–2.35)	1.50 (1.05–2.15)	1.22 (0.84–1.77)
High (31.30–44.92)	4.09 (2.97–5.64)	3.71 (2.68–5.15)	2.90 (2.02–4.15)
*P* for trend	<0.001	<0.001	<0.001

*OR, odds ratio; CI, confidence interval; aOR, adjusted odds ratio. Model 1 adjusts for: none. Model 2 adjusts for: maternal ethnicity, maternal education, maternal occupation, and maternal income. Model 3 adjusts for: maternal ethnicity, maternal education, maternal occupation, maternal income, maternal body mass index, parity, folic acid supplementation, multivitamins supplementation, and medication use*.

### Threshold Effect Analysis of Maternal Age on Spontaneous Abortion

**Figure 2:**
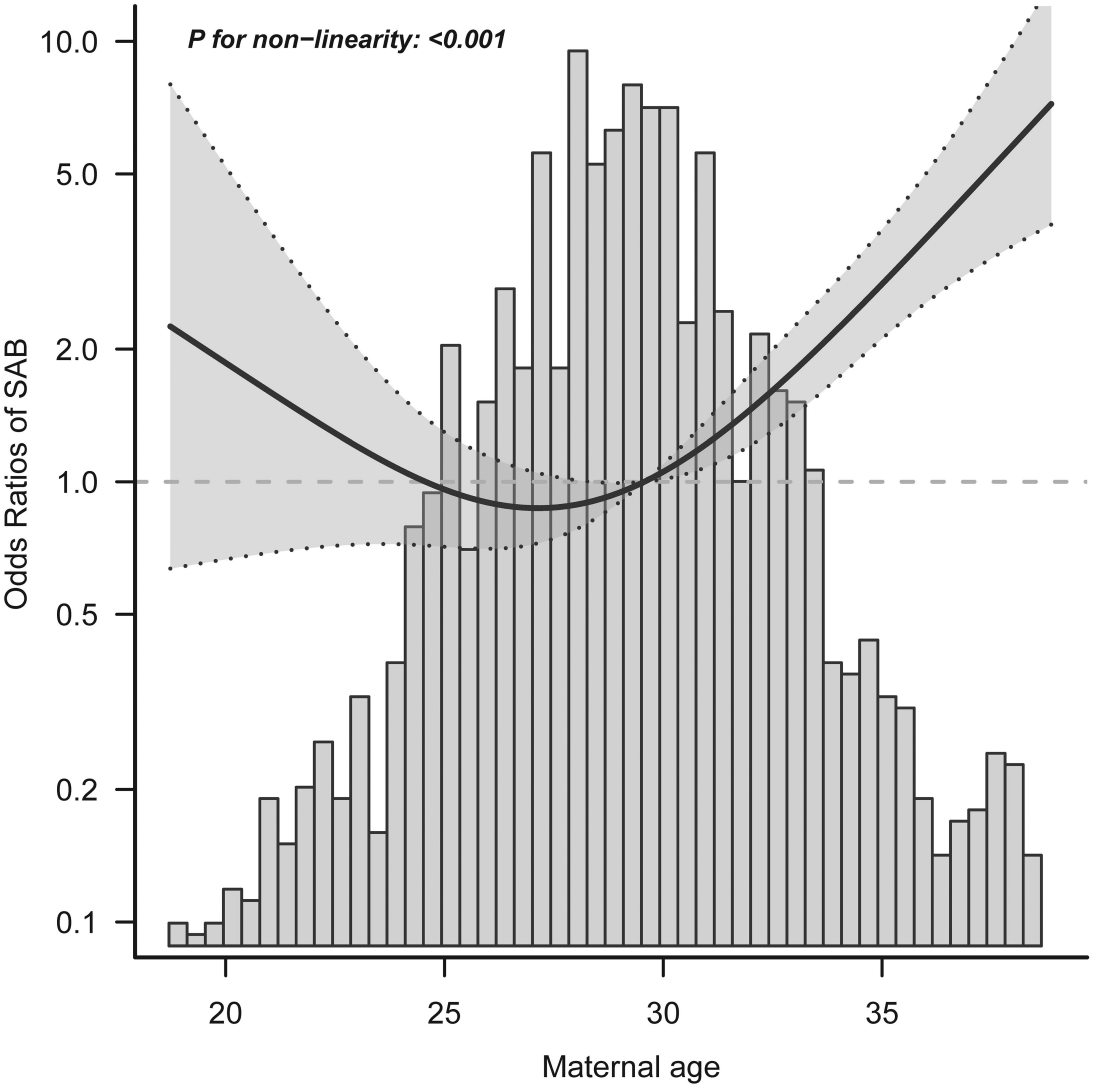
Exposure-response relationship between maternal age and spontaneous abortion. SAB, spontaneous abortion; CI, confidence interval. Data were fit by a logistic regression model based on restricted cubic splines. Maternal age was entered as continuous variable. Data were adjusted for maternal ethnicity, maternal education, maternal occupation, maternal income, maternal body mass index, parity, folic acid supplementation, multivitamins supplementation, and medication use. The gray area represents the 95% CI.

We found that the relationship between maternal age and spontaneous abortion was non-linear (after adjusting for maternal ethnicity, maternal education, maternal occupation, maternal income, maternal BMI, parity, folic acid supplementation, multivitamins supplementation, and medication use) (Figure 2). Using a 2-piecewise linear regression model, we further identified the inflection point as 29.68 years. To the left of the inflection point, when maternal age was ≤ 30 years, an increase in maternal age was not significantly associated with risk of spontaneous abortion (aOR = 0.97; 95% CI: 0.90–1.06; *P* = 0.542). To the right of the inflection point, when maternal age was >30 years, each 1-year increase in maternal age was significantly associated with increased risk of spontaneous abortion (aOR = 1.25; 95% CI: 1.18–1.31; *P* < 0.001) (Table 3). After adjusting for all variables except maternal age, the results remained stable (Supplementary Tables S2, S3).

**Table 3:** Threshold effect analysis of maternal age on spontaneous abortion.

**Variables**	**aOR (95% CI)**	***P-*value**
Maternal age (continuous), years	1.16 (1.12–1.20)	0.001
Turning point	29.68	
**Maternal age (two-piece-wise linear regression), years**		
≤ 30	0.97 (0.90–1.06)	0.542
>30	1.25 (1.18–1.31)	0.001
Log-likelihood ratio test		<0.001

*aOR, adjusted odds ratio; CI, confidence interval. Adjusts for: maternal ethnicity, maternal education, maternal occupation, maternal income, maternal body mass index, parity, folic acid supplementation, multivitamins supplementation, and medication use*.

### Stratified Analyses

**Figure 3:**
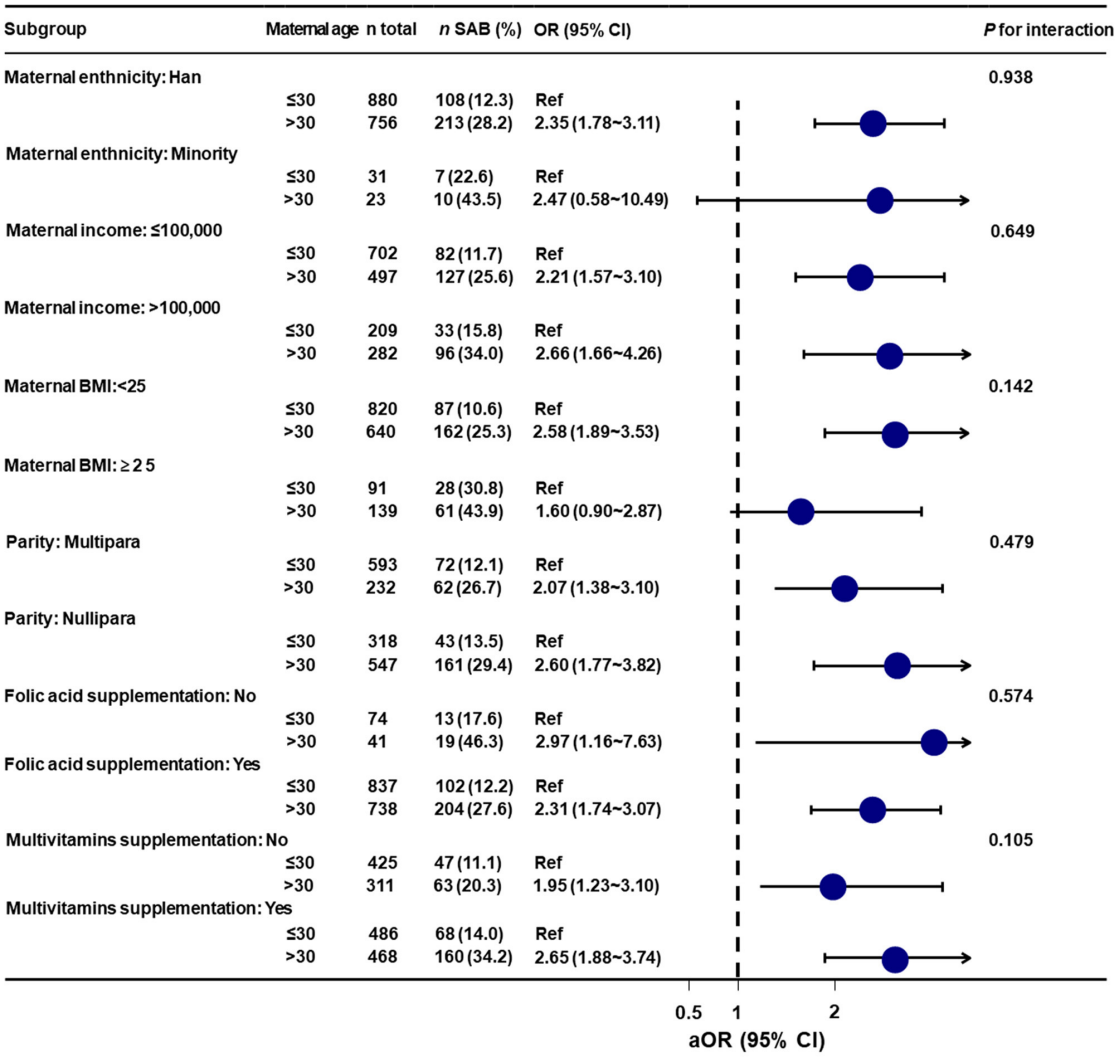
Stratified analyses of the association between maternal age and spontaneous abortion. aOR, adjusted odds ratio; Ref, reference; BMI, body mass index; SAB, spontaneous abortion; CI, confidence interval. Data were fit by a logistic regression model based on restricted cubic splines. Maternal age was entered as continuous variable. Data were adjusted for maternal ethnicity, maternal education, maternal occupation, maternal income, maternal body mass index, parity, folic acid supplementation, multivitamins supplementation, and medication use.

Stratified analyses showed that no significant associations were observed among all of the above subgroups (*i.e.*, maternal ethnicity, maternal income, maternal BMI, parity, folic acid supplementation, and multivitamins supplementation) (all interaction test *P* > 0.05) (Figure 3).

## Discussion

In the current study, we found a J-shaped relationship between maternal age and spontaneous abortion risk, after adjusting for multiple covariates. Further, we found that the optimal threshold of maternal age was 29.68 years. The aOR and 95% CI of risk of spontaneous abortion per 1 year increase in maternal age were 0.97 (0.90–1.06) on the left side of the turning point and 1.25 (1.28–1.31) on the right side. Importantly, none of the covariates studied modified the association between maternal age and spontaneous abortion. Overall, the threshold effect analysis shows that when maternal age lower than 30 years, there is a significantly lower risk for spontaneous abortion in China.

### Comparisons With Previous Studies

Our findings were consistent with most of previous studies that the association between maternal age and spontaneous abortion follows a J-shaped curve (the association trend between spontaneous abortion and maternal age was consistent with that between miscarriage and maternal age) (4, 17, 44, 45). The majority of the previous research reports a positive association between advanced maternal age and spontaneous abortion (17, 46, 47). However, the ideal maternal age has still not been clearly elucidated. We also observed that maternal age >30 years was significantly positively associated with incidence of spontaneous abortion, which was consistent with many of the previous studies. Authors including Linnakaari et al. (19), Rossen et al. (4), and Magnus et al. (17) claimed that maternal age >30 years was a threshold age for spontaneous abortion. However, Quenby et al. (16) found that the higher risk of spontaneous abortion came at a maternal age <20 years old. Feodor et al. (18) found that the risk of spontaneous abortion in pregnant women was significantly higher when maternal age was over 40 years old. Two small-sample studies in China found that pregnant women who were 32 years old or older showed a stronger risk association with spontaneous abortion (20, 21).

The results of this study were consistent with patterns from real-world research, which integrates host factors as well as social and environmental elements (48). First, host factors such as overweight and obesity have rapidly become prevalent among advanced age pregnant women worldwide (49). In America, more than half (50.04%) of pregnant women are overweight and obesity (50). In the Chinese population, 1 in 4 pregnant women suffers from overweight or obesity (51). It is also possible that genetic effects are more evident at older maternal age, due to the accumulation of exposures to environmental risk factors for spontaneous abortion with age (52). Second, older pregnant women are more vulnerable to pregnancy-specific anxiety (53). The literature demonstrates that social support from partners, family members and friends are beneficial to the health of pregnant women (54). However, many pregnant women receive less support during their pregnancies (55). Third, the cumulative effect of environmental factors may play a crucial role in advanced maternal age's effect on spontaneous abortion (56). Previous studies found correlations between ambient air pollutant levels (57), exposure to Bisphenol A (58), and exposure to heavy metals such as cadmium, lead, and mercury (59) and spontaneous abortion.

There are several reasons that help illustrate the mechanism of the association between advanced maternal age (>30 years old) and spontaneous abortion: (a) an age-related decline in reproductive capacity is due to the gradual decline in ovarian reserves and oocyte integrity (60); (b) more frequent chromosome separation errors lead to oocyte cell aneuploidy (47), which is believed to be the main cause of a mother's age-related spontaneous abortion; (c) there are multiple copies of the mitochondrial genome, but as we get older, different mtDNA copies will accumulate more and more mutations, so the risk of getting mutations is greater with age (61). In addition, mothers of advanced age are more likely to develop a medical condition during pregnancy, such as obesity, anemia, and diabetes (62). Still, the etiology of most cases of spontaneous abortion remains unknown and requires further investigation.

### Strengths and Limitations

Our study has a number of strengths. First, we used the generalized additive model to investigate the non-linear relationship between maternal age and spontaneous abortion. This model can not only handle non-parametric smoothing and fit regression splines, but can also help us better discover the true relationship between exposure and results. Second, the current study is an observational study, including inevitable potential confounding. According to the recommendation of the STROBE statement, we used strict statistical adjustments to minimize residual confounding. Additionally, we excluded participants with known surgery abortions and medical abortions, which might affect our results. Finally, stratified analyses demonstrated that our results were robust.

Several limitations cannot be overlooked. First, since our participants were from four hospitals, the representativeness of our study sample may be limited compared to the national statistics. Further study based on larger sample is warranted to confirm our findings. Second, although we have controlled for important epidemiological and clinical covariates in the analysis, we cannot rule out the possibility of other residual confounding. For example, this study did not consider maternal infection factors, clinical laboratory examination index factors, emotional factors, etc. Finally, our findings are not causal, and we need to be cautious when interpreting the results. An additional and important limitation is that likely many cases of spontaneous abortion are missing as they have gone unmeasured.

## Conclusion

The relationship between maternal age and spontaneous abortion is J-shaped. Advanced maternal age (>30 years old) was significantly associated with increased risk of spontaneous abortion. None of the covariates studied modified the association between maternal age and spontaneous abortion.

## Data Availability Statement

The original contributions presented in the study are included in the article/Supplementary Material, further inquiries can be directed to the corresponding author/s.

## Ethics Statement

The studies involving human participants were reviewed and approved by the Ethical Committee of Beijing Obstetrics and Gynecology Hospital, Capital Medical University (No. IEC-C-29-V02-FJ1). The patients/participants provided their written informed consent to participate in this study.

## Author Contributions

WY had full access to all the data of the study and takes responsibility for the integrity and accuracy of the data analysis. MZ, B-YY, and YS: statistical analysis. WY, CY, G-HD, RL, MZ, B-YY, and YS: concept and design. MZ, B-YY, YS, WY, CY, G-HD, ZQ, PX, HA, XZ, and ZZ: acquisition, analysis, or interpretation of data. MZ, B-YY, YS, WY, CY, and G-HD drafting of the manuscript. MZ, B-YY, YS, ZQ, PX, HA, XZ, ZZ, RL, G-HD, CY, and WY: critical revisions of the manuscript for important intellectual contents, administrative, technical, and material support. WY, CY, MZ, and YS obtained funding. WY, CY, G-HD, and RL: supervision. All authors contributed to the article and approved the submitted version.

## Funding

This work was supported by the National Key Research and Development Program of China (Nos: 2016YFC1000101 and 2019YFC1005100), China Postdoctoral Science Foundation (No: 2020TQ0207). Postdoctoral Foundation provide by Beijing Obstetrics and Gynecology Hospital, Capital MedicalUniversity.

## Conflict of Interest

The authors declare that the research was conducted in the absence of any commercial or financial relationships that could be construed as a potential conflict of interest.

## Publisher's Note

All claims expressed in this article are solely those of the authors and do not necessarily represent those of their affiliated organizations, or those of the publisher, the editors and the reviewers. Any product that may be evaluated in this article, or claim that may be made by its manufacturer, is not guaranteed or endorsed by the publisher.
