# Supplementary material for: Non-linear Relationship of Maternal Age With Risk of Spontaneous Abortion: A Case-Control Study in the China Birth Cohort
Source: Front Public Health. 2022 Jul 14;10:933654. doi: 10.3389/fpubh.2022.933654 (PMC9330030; doi:10.3389/fpubh.2022.933654)
Supplement: Supplementary file 1 [file Data_Sheet_1.docx]

**TABLE S1 |** Univariate logistic regression analysis for the association between maternal age and spontaneous abortion.

| **Variables** | **Statistics** | **OR (95%CI)** |
| --- | --- | --- |
| **Maternal age*, years** | 29.85 ± 4.15 | 1.18 (1.15, 1.22) |
| **Maternal BMI*, kg/m^2^** | 21.61 ± 3.40 | 1.13 (1.10, 1.17) |
| **Maternal ethnicity*, *n* (%)** |  |  |
| Han | 1636 (96.80%) | Ref |
| Minority | 54 (3.20%) | 1.88 (1.05, 3.39) |
| **Maternal education*, *n* (%)** |  |  |
| College | 688 (40.71%) | Ref |
| Below college | 875 (51.78%) | 0.83 (0.64, 1.06) |
| College graduate | 127 (7.51%) | 1.69 (1.11, 2.57) |
| **Maternal occupation*, *n* (%)** |  |  |
| Manual | 810 (47.93%) | Ref |
| Non-manual | 447 (26.45%) | 0.80 (0.60, 1.07) |
| Unemployed | 433 (25.62%) | 0.66 (0.49, 0.90) |
| **Maternal secondhand smoking, *n* (%)** |  |  |
| No | 1479 (87.51%) | Ref |
| Yes | 205 (12.13%) | 1.07 (0.75, 1.54) |
| Missing | 6 (0.36%) | 2.02 (0.37, 11.10) |
| **Maternal drinking, *n* (%)** |  |  |
| No | 1610 (95.27%) | Ref |
| Yes | 79 (4.67%) | 1.10 (0.64, 1.91) |
| Missing | 1 (0.06%) | 0.00 (0.00, inf.) |
| **Maternal income*, yuan, *n* (%)** |  |  |
| 50000-100000 | 713 (42.19%) | Ref |
| >100000 | 491 (29.05%) | 1.76 (1.33, 2.33) |
| <50000 | 486 (28.76%) | 1.11 (0.82, 1.50) |
| **Parity*, *n* (%)** |  |  |
| Nullipara | 825 (48.82%) | Ref |
| Multipara | 865 (51.18%) | 1.59 (1.25, 2.03) |
| **Conception method, *n* (%)** |  |  |
| Natural conception | 1608 (95.15%) | Ref |
| Assisted reproduction | 82 (4.85%) | 1.22 (0.72, 2.07) |
| **Folic acid supplementation*, *n* (%)** |  |  |
| First trimester of pregnancy | 991 (58.64%) | Ref |
| Before pregnancy | 584 (34.56%) | 1.22 (0.94, 1.57) |
| No | 115 (6.80%) | 1.73 (1.11, 2.68) |
| **Multivitamin supplementation*, *n* (%)** |  |  |
| First trimester of pregnancy | 680 (40.24%) | Ref |
| Before pregnancy | 274 (16.21%) | 1.82 (1.33, 2.50) |
| No | 736 (43.55%) | 0.68 (0.51, 0.89) |
| **Medicine use*, *n* (%)** |  |  |
| No | 1015 (60.06%) | Ref |
| Yes | 675 (39.94%) | 1.33 (1.05, 1.70) |

*Abbreviations: BMI, body mass index; OR, odds ratios; CI, confidence intervals;*

** Significant results of univariate analysis are adjusted as confounders.*

**TABLE S2 |** Multivariate logistic analysis for the association of maternal age with spontaneous abortion.

| **Variables** | **Model 1 OR (95% CI)** | **Model 2 aOR (95% CI)** | **Model 3 aOR (95% CI)** |
| --- | --- | --- | --- |
| **Maternal age (continuous), years** | 1.18 (1.15, 1.22) | 1.18 (1.14, 1.22) | 1.16 (1.12, 1.20) |
| **Maternal age (tertile), years** |  |  |  |
| Low (18.73-28.01) | Reference | Reference | Reference |
| Middle (28.02-31.29) | 1.66 (1.17, 2.35) | 1.50 (1.05, 2.15) | 1.23 (0.85, 1.79) |
| High (31.30-44.92) | 4.09 (2.97, 5.64) | 3.71 (2.68, 5.15) | 2.96 (2.06, 4.26) |
| ***P* for trend** | <0.001 | <0.001 | <0.001 |

*Abbreviations: OR, odds ratios; CI, confidence intervals; aOR, adjusted odds ratios.*

*Model 1 adjusts for: none.
Model 2 adjusts for: maternal ethnicity, maternal education, maternal occupation, and maternal income.*

*Model 3 adjusts for:* *maternal ethnicity, maternal education, maternal occupation, maternal income, maternal body mass index, maternal secondhand smoking, maternal drinking, parity, conception method, folic acid supplementation, multivitamins supplementation, and medication use.*

**TABLE S3 |** Threshold effect analysis of maternal age on spontaneous abortion.

| **Variables** | **aOR (95% CI)** | ***P*-value** |
| --- | --- | --- |
| **Maternal age (continuous), years** | 1.16 (1.12, 1.20) | <0.001 |
| **Turning point** | 29.78 |  |
| **Maternal age (two-piece-wise linear regression), years** |  |  |
| ≤30 | 0.98 (0.91, 1.07) | 0.658 |
| >30 | 1.25 (1.19, 1.32) | <0.001 |
| Log-likelihood ratio test |  | 0.001 |

*Abbreviations: CI, confidence intervals; aOR, adjusted odds ratios;*

*Adjusts for: maternal ethnicity, maternal education, maternal occupation, maternal income, maternal body mass index, maternal secondhand smoking, maternal drinking, parity, conception method, folic acid supplementation, multivitamins supplementation, and medication use.*
